# Efficient biosorption of hexavalent chromium from water by modified arecanut leaf sheath

**DOI:** 10.1016/j.heliyon.2022.e09283

**Published:** 2022-04-19

**Authors:** Bishnu Datt Pant, Deepa Neupane, Dasu Ram Paudel, Prakash Chandra Lohani, Surendra Kumar Gautam, Megh Raj Pokhrel, Bhoj Raj Poudel

**Affiliations:** a Department of Chemistry, Tri-Chandra Multiple Campus, Tribhuvan University, Kathmandu 44600, Nepal; b Department of Chemistry, Amrit Campus, Tribhuvan University, Kathmandu 44600, Nepal; c Central Department of Chemistry, Tribhuvan University, Kathmandu 44618, Nepal

**Keywords:** Cr(VI), Biosorbent, CALS, Adsorption isotherms, Kinetics

## Abstract

In this work, the excision of hexavalent chromium (Cr(VI)) was studied from an aqueous solution using the chemically modified arecanut leaf sheath (CALS) as a novel bio-adsorbent. The as-prepared adsorbent was characterized by using instrumental methods including Fourier-transform infrared spectroscopy (FTIR), scanning electron microscopy (SEM), and energy dispersive spectroscopy (EDS). The effect of several factors, including solution pH, contact time, and sorbent dosages were examined to identify the optimum condition for the sorption ability. The optimal pH of Cr(VI) biosorption was 2.0, and equilibrium was reached in 150 min. Adsorption was shown to be pseudo-second-order in kinetic investigations, and the Langmuir isotherm with maximal adsorption efficiency was determined as 109.89 mg/g. The spent biosorbent can be easily regenerated and reused. For the biosorption of oxyanions of chromium, both electrostatic attraction and ligand exchange mechanism play critical roles. From the results, the CALS appears to be a potential low-cost effective sorbent to remove Cr (VI) from water.

## 1 Introduction

Water is the foremost substance on earth and all plants and animals must require water to survive. Nowadays, water pollution is a serious problem in the environment, and its pollution from anthropogenic sources is common, which deteriorates the quality of water (Rai et al., 2018). Among all pollutants, heavy metals possess major worries due to their highly toxic, imperishable, and steady nature. Hexavalent chromium, cadmium (Cd), arsenic (As), mercury (Hg), nickel (Ni), cobalt (Co), etc are the major toxic heavy metal pollutants (Rathore et al., 2017; Rai et al., 2021) and their pollution is not only detrimental to the surroundings, but they also further cause a serious problem for human health. Amidst harmful heavy metals, hexavalent chromium is examined as a major water toxin. Chromium metal is often found in wastewaters from natural and manmade sources. Amongst these, the major sources are the different industries, for example, leather tanneries, textile industries, electroplating industries, paint and pigment industries (Suganya et al., 2019; Mahmoud, 2020). They are primarily found in two oxidation states: Cr(III) and Cr(VI), with varying concentrations from 10 to 100 mg/L (Vunain et al., 2021). The species containing Cr (VI) have additional solubility and 500-fold greater toxicity compared to Cr (III) species. It is proven that hexavalent chromium (Cr) causes dermatitis inflammation, vomiting, epigastric pain, bleeding, diarrhea, and cancer in the human body (Yi et al., 2017; Saha et al., 2011). Its higher toxicity than trivalent chromium is due to its high solubility, mobility, as well as easy reduction (Parlayici and Pehlivan, 2019; Chen et al., 2018). Hexavalent chromium affects humans by oxidizing the DNA building block and some other protein molecules found in humans (Bansal et al., 2009). Chromium is highly toxic in water, and as it bioaccumulates in the body by entering food chains, it causes toxicity in plants and other lifeforms (Costa and Klein 2006). Cr(VI) is more soluble, toxic, and classified as a “Class A” human carcinogen, mutagen, and teratogen (Rathore et al., 2021). According to mortal consequences on human health, the maximum toxicity levels for hexavalent chromium in drinking water and industrial emissions are 0.05 mg/L and 0.25 mg/L respectively (Zhang et al., 2021; Suganya et al., 2019) so, Cr (VI) removal from sewer water is necessary.

Heavy metals damage water resources and soil through wastewater and solid waste disposal because of their usage in various industrial applications such as tanning, metallurgy, plating, and metal polishing (Demiral et al., 2008). To minimize heavy metal ion content in wastewater, many techniques have been researched and used. Precipitation, ion exchange, adsorption on activated carbon, membrane processing, and electrolytic processes are examples of these techniques (Marghaki et al., 2020; Ye et al., 2019; Bhujel et al., 2021). Most of the methods were determined to be expensive. They mostly require large funds and functional costs and cause alternative contamination of water resources (Gupta and Babu, 2009).

Recent research has focused on agricultural wastes, which are relatively cheap and abundant, and contain a variety of polymeric substances such as cellulose, hemicelluloses, pectin, lignin, and proteins, all of which can bind heavy metals significantly. Various agricultural wastes were used to eliminate the metal pollutant by the process of adsorption. These are green moringa leaves (Timbo et al., 2017), nutshells (Pehlivan and Altun, 2008), sugarcane bagasse (Garg et al., 2007; Bhatt et al., 2018), coconut coir (Shen et al., 2010), and sweet lime peels (Adhikari et al., 2017). Recently functionalized multiwall carbons nanotubes modified with different stem bark extracts such as Pentaclethra macrophylla (Amaku et al., 2021), have been used for the adsorption of Cr(VI).

Arecanut leaf sheath is rich in cellulose content that is involved in metal binding (Poddar et al., 2016). It is also commonly accessible in eastern regions of Nepal. It is commonly dumped as waste residue and is readily accessible for free or a very low price. As a result, it might be employed as a low-cost adsorbent for removing Cr(VI) from water. Due to a lack of an adequate economical material for the removal of Cr (VI) ions, we investigated the fabrication of low-cost adsorbents utilizing arecanut leaf sheath since financial appropriateness is a necessity for the absorption of chromium from water in rural regions. Arecanut leaf sheath is commonly accessible as bio-waste in many places, contains various surface functional groups, and does not discharge soluble pollutants into the treated water, making it an ideal candidate for our investigation. Furthermore, acid modification may promote Cr(VI) ion sorption because the acid treatment of raw biomass may offer a high number of active sites that can interact chemically with Cr (VI), thereby improving chemisorption. To the foremost of our knowledge, there is no study reported in the literature of CALS for the adsorptive removal of Cr(VI). Therefore, CALS is proposed as a novel adsorbent for the removal of Cr(VI) from the aqueous solution.

A major objective of this study was to use the most abundant and cheap agricultural waste obtained from the arecanut tree and modify it chemically to get the CALS, which was investigated to remove hexavalent chromium. Adsorbents were characterized by several characterization techniques, namely FTIR, XRD, SEM, and EDS. The outcomes of different factors, like solution pH, interaction time, biosorbent dosages, and Cr(VI) concentration, were investigated. The desorption study determined the regeneration and reusability of the adsorbent.

## 2 Experimental

### 2.1 Chemicals

Only analytical grade chemical reagents were used, and they have not been purified additionally. A stock solution of 1000 mg/L potassium dichromate was prepared in a 0.1N nitric acid solution.1 mL of stock potassium dichromate solution equals 1000 μg HCrO_4_^−^ in the form of Cr(VI). Working solutions were prepared by dilution method in 0.1N nitric acid. 0.1 M HCl and 0.1 M NaOH solution were used to adjust the pH and 0.1 M 2-[4-(2-hydroxymethyl)-1-piperazinyl] ethane sulfonic acid was used as a buffering agent. The initial and equilibrium concentration of Cr(VI) in the solution was determined by an Inductively Coupled Plasma-Mass Spectrometer (ICP-MS) (Agilent Technologies, 7900 ICP-MS, Santa Clara, CA, USA) with argon plasma.

### 2.2 Adsorbent preparation

**Scheme 1:**
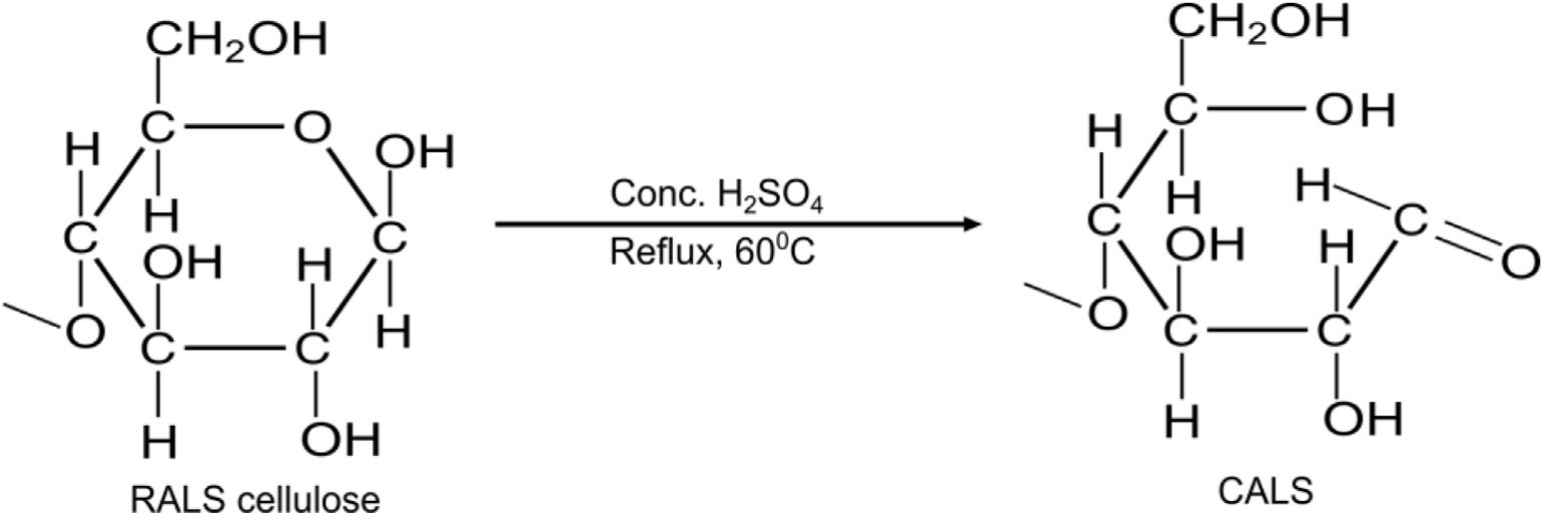
Synthesis mechanism of CALS.

The arecanut leaf sheath (ALS) was collected from the plain region of Jhapa, Nepal. The collected leaf sheath was rinsed with deionized (DI) water and was dried in sunlight for a week which was further dried in the oven at 70 °C for 24 h. The sample was then ground by a mechanical grinder and then sieved to 80 mesh and is referred to as raw arecanut leaf sheath (RALS). The chemical composition of ALS constitutes 66.08% cellulose, 7.40% hemicellulose, 5.06% waxy matters, 1.15% pectic matters, and 0.72% aqueous extract (Poddar et al., 2016). The RALS powder was refluxed with concentrated sulfuric acid 98% (density = 1.84 g/cm^3^, Molarity = 18.4 M) in a 1:3 (w/v) ratio and allowed to immerse for 5 h at 60 °C. The arecanut tree can be found in abundance in eastern areas of Nepal. Arecanut leaf sheath (ALS) biopolymer is rich in cellulose content, which helps with metal binding. Furthermore, acid modification can help to provide the right conditions for the cellulose ring to open. It also raises the surface area of the material and causes a lot of microporosity (Homagai et al., 2010). It is expected to activate hydroxy surface functional groups contained in lignin, cellulose, hemicelluloses, and polyphenol in the RALS at moderate temperature. The reaction mechanism for the synthesis of CALS is shown in Scheme 1 (Ioelovich, 2012). After that, the excess acid and any other soluble substances were detached by washing with DI water till neutrality and further desiccated at 60 °C for 6 h. Thus, prepared finely powdered material is referred to as chemically modified arecanut leaf sheath (CALS) and was kept in air-tight containers.

### 2.3 Characterization of adsorbents

FTIR spectroscopy (IR AFFINITY-1 Shimadzu, Kyoto, Japan) was used to identify the functional groups of the adsorbents. Morphology and elemental composition of adsorbents were analyzed by SEM images and EDS (JEOL JSM–6700F Ltd., Tokyo, Japan) equipped with X-ray Energy Dispersive Spectrometer. X-ray diffraction (XRD) was used to analyze the crystallinity of the adsorbent by Rigaku Ultima IV X-ray diffractometer (Rigaku Co., Tokyo, Japan). Zeta potential measurements were used to explore the superficial charge characteristic of the biosorbent using a Zeta potential analyzer (HORIBA Scientific SZ-100).

### 2.4 Biosorption experiments

Adsorption studies for both raw and modified arecanut leaf sheath were carried out by taking 40 mL of known initial concentration of Cr(VI) and 50 mg of biosorbent in a 100 mL volumetric flask. The mixture of adsorbent and metal solution was shaken for 12 h at 150 rpm in a shaker at room temperature (298K) to confirm equilibrium. The equilibrated solution was filtered, and filtrates were utilized for the analysis of the residual concentration of chromium ions. The concentration of chromium before and after sorption was analyzed by ICP-MS.

In batch adsorption studies the effect of pH of the solutions, initial concentration of Cr(VI) ion, and contact time was also studied. The adsorption capacity at equilibrium *q*_e_ (mg/g) and percentage removal (R_e_ %) was identified by the specified Eq. (1) and Eq. (2), respectively (Poudel et al., 2021).

(1) $$q_{e}=\frac{\left(C_{o}-C_{e}\right)V}{m}$$

(2) $$R_{e}\%=\frac{\left(C_{o}-C_{e}\right)}{C_{o}}\times 100$$

where *C*_*o*_ is the initial concentration (mg/L), *C*_*e*_ is the equilibrium time concentrations of Cr(VI) (mg/L), *m* is the adsorbent mass (g), and *V* is the volume of the solution (L)

#### 2.4.1 Isotherm study

The monolayer adsorption on the surface of the biosorbent is described by the Langmuir isotherm model. This model is represented by non-linearly using Eq. (3), and linearly using Eq. (4) as follows (Langmuir, 1916),

(3) $$q_{e}=\frac{q_{\max}\cdot K_{\text{L}}\cdot C_{e}}{1+K_{L}\cdot C_{e}}$$

(4) $$\frac{C_{e}}{q_{e}}=\frac{1}{q_{\max}+K_{L}}+\frac{C_{e}}{q_{\max}}$$

where, *K*_*L*_ (L/mg) is the Langmuir constant, and *q*_*max*_ (mg/g) is the maximum adsorption capacity.

Langmuir isotherm quality can be determined by the magnitude of dimensionless constant (*R*_*L*_) called separation factor by using Eq. (5). It determines the nature of the adsorption isotherm (Wang and Guo, 2020). The adsorption process is favorable (0 < R_L_ < 1), unfavorable (R_L_> 1), linear (R_L_ = 1) or Irreversible (R_L_ = 0).

(5) $$R_{L}=\frac{1}{1+\left({\text{K}}_{L}C_{o}\right)}$$

Multilayer adsorption over the heterogeneous surface is determined by the Freundlich isotherm and the model is expressed using non-linear Eq. (6) and linear Eq. (7) as follows (Freundlich, 1906),

(6) $$q_{e}=K_{F}{\left(C_{e}\right)}^{1/n}$$

(7) $$\log\;q_{e}=\log\;K_{F}+\left(\frac{1}{n}\right)\log\;C_{e}$$

where *K*_*F*_ [(mg/g) (L/mg)^1/n^] is the Freundlich constant indicates adsorption capacity and *n* is Freundlich exponent indicates an adsorption intensity.

#### 2.4.2 Adsorption kinetics

Kinetics of adsorption was identified by using pseudo-first-order and pseudo-second-order models which gives the insight into controlling mechanism of the adsorption of Cr(VI) on the adsorbent. Pseudo first order linear equation (Lagergren, 1998) is expressed by Eq. (8) as,

(8) $$\log\left(q_{e}-q_{t}\right)=\log\;q_{e}-\frac{K_{1}}{2.303}t$$

where *q*_*t*_ is the sorption capacity at time *t* (mg/g) and *k*_*1*_ (mg/g.min) is the rate constant of pseudo first order. Pseudo second order linear equation (Ho, 2006) is expressed by Eq. (9) as,

(9) $$\frac{t}{q_{t}}=\frac{1}{k_{2}q_{e}^{2}}\frac{1}{q_{e}}t$$

where *K*_*2*_ (g mg^−1^ min^−1^) is the rate constant of pseudo second order model.

To elucidate the diffusion mechanism, the kinetics results were evaluated using the Weber-Morris's intraparticle diffusion model (Weber and Morris 1963), which is expressed by Eq. (10):

(10) $$q_{t}=\left(k_{id}\times t^{0.5}\right)+C$$

where k_id_ (mg/g min^−0.5^) is the intraparticle diffusion rate constant, and c (mg/g) is the Weber-Morris equation intercept, which indicates the thickness of the boundary layer, i.e. the bigger the value of C, the stronger the boundary layer effect.

#### 2.4.3 Desorption and reusability of CASL

Regeneration of the biosorbent is a crucial aspect to evaluate its economic feasibility. Reusability of the adsorbent was done following reported procedures (Poudel et al., 2020) aiming to accomplish economic enhancement of the adsorption by renewing and reusing the material. Desorption studies were done by mixing 250 mg of CALS with 250 mL of chromium (25 mg/L) at pH 2.0 for 4 h at room temperature. The % desorption (% D) of Cr(VI) is expressed using Eq. (11),

(11) $$\%D=\frac{D_{amount}}{A_{amount}}\times 100$$

where A_amount_ is the adsorbed amount (mg/g) and D_amount_ is the desorbed amount (mg/g) of Cr(VI) ions.

## 3 Results and discussion

### 3.1 Characterizations of adsorbents

**Figure 1:**
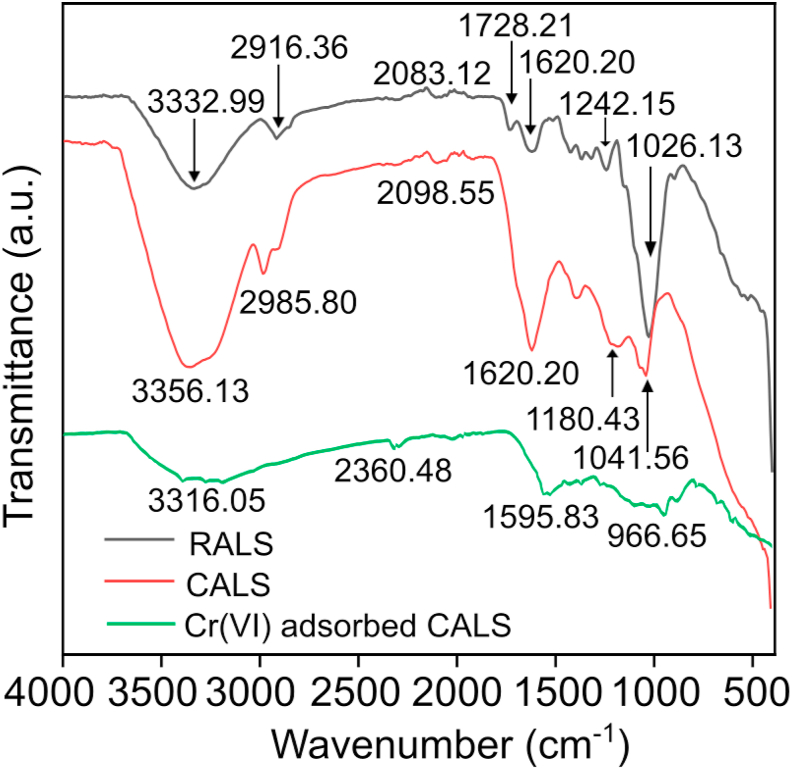
FTIR spectra of RALS, CALS, and Cr(VI) adsorbed CALS.

In the FTIR spectra of RALS, a broad and strong band at 3332.99 cm^−1^ is stretching vibration of (OH) groups of cellulose, hemicelluloses, and lignin. This peak widens and shifts to 3356.13 cm^−1^ in the case of CALS which may be due to the ring-opening of cellulose molecules and the presence of many OH groups. A peak at 2916.36 cm^−1^in RALS is because of the C-H stretching vibration and it shifted to 2985.80 cm^−1^ for the CALS (Figure 1). Further, a peak at 1728.21 cm^−1^ in RALS is assigned to the C=O stretching vibration of the carbonyl group and a peak at 1026.13 cm^−1^ is attributed to the stretching vibration of primary alcohol (Sun et al., 2014). The FTIR spectra of the CALS sample showed the switch in intensity and frequency range of hydroxyl and carboxyl stretches. Additionally, there are the emergence of novel peaks and vanishing of few peaks in the case of CALS as compared to the RALS. The Cr(VI) adsorbed CALS spectra showed the peak at 3316.05 cm^−1^ (–OH stretching vibration) is broader and of reduced intensity compared to CALS which is likely because of the participation of –OH groups to adsorb the chromium. Moreover, the sharp peaks that were present in CALS have disappeared and an extra peak has appeared at 2360.48 cm^−1^. The switch in intensity and emergence of some peaks are credible to the coordination of Cr(VI) ions to the functional group, while the disappearance of a peak suggests the participation of chemical reactions, which leads to decomposing the chemical bonds. All the above factors suggested the successful chemical modification of RALS into CALS and there is strong adsorption of chromium onto the CALS.

**Figure 2:**
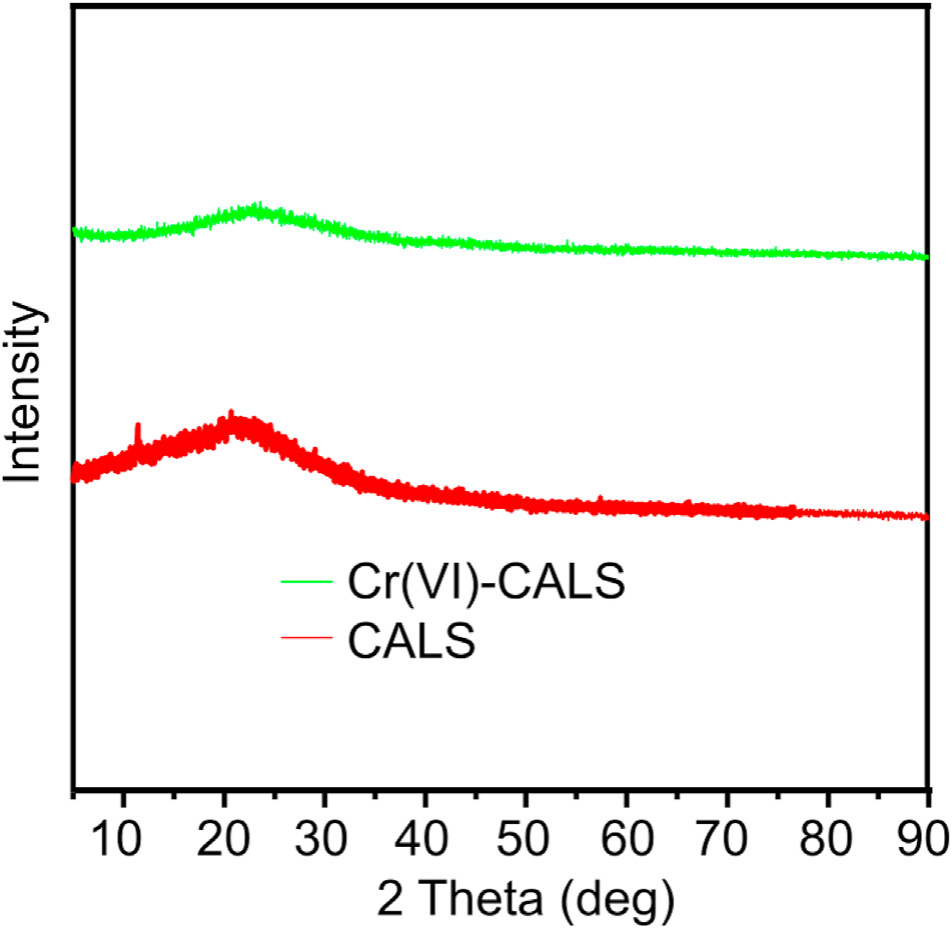
EDAX color mapping illustrating the elemental distribution of SPP@Zr (a) before adsorption and (b) after adsorption.

XRD spectra of raw CALS and Cr(VI)-CALS are given in Figure 2 and it was found that there were no sharp peaks in both cases as of the crystalline materials. Adsorbents were predominantly found in an amorphous nature since there was no calcination during the chemical modification to synthesize the CALS.

**Figure 3:**
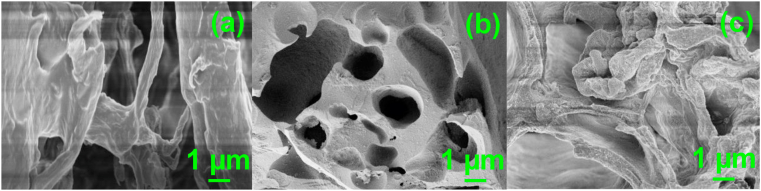
SEM images of (a) RALS (b) modified CALS and (c) CALS after Adsorption.

**Figure 4:**
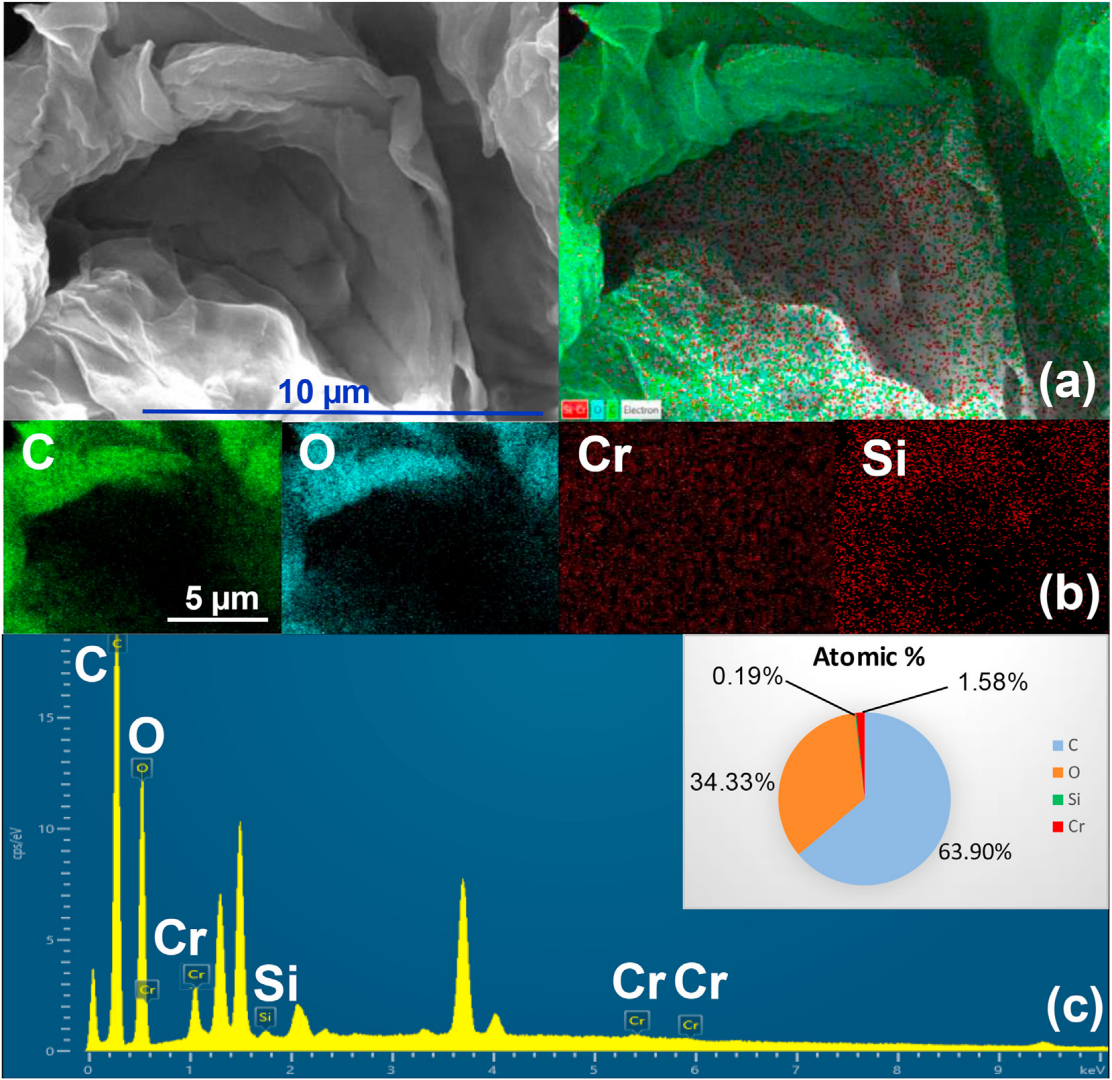
(a) EDS electron and layered image, (b) EDS mapping images of all overlapping elements and (c) EDS atomic percentage of all elements of Cr(VI) adsorbed CALS.

Figure 3 shows the SEM image of RALS, CALS, and CALS after adsorption. RALS surface structure was observed, and it is found to be smooth, homogenous with fewer pores and cavities. The CALS showed a rough, heterogenous surface with many pores and cavities indicated the modification of RALS to CALS. During the chemical modification process, rough and porous surfaces appeared which might be the removal of smooth and waxy materials from the surface as such as sugar molecules and limonene. Further, in the case of chromium adsorbed CALS, the roughness of the surface was decreased because of the adsorption of Cr(VI) ions. The adsorption of chromium was established by EDS analysis and elemental mapping of CALS after chromium adsorption (Figure 4). The elemental composition of Cr(VI) adsorbed CALS was evaluated which showed the presence of chromium with a decent elemental composition.

**Figure 5:**
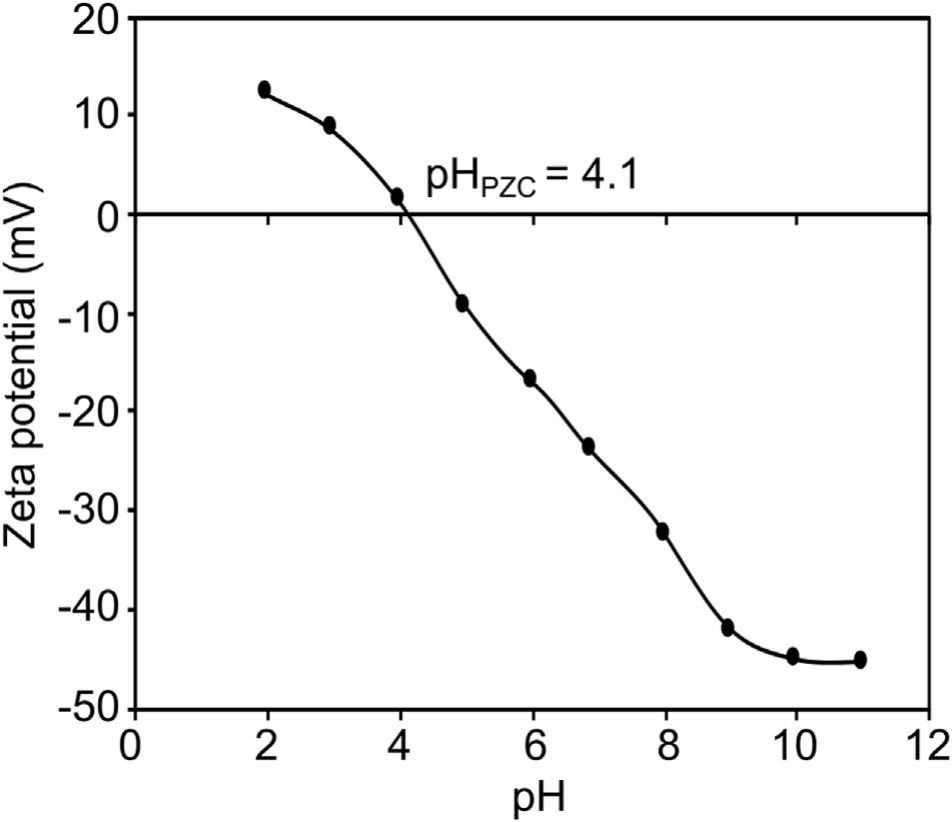
Zeta potential of CALS.

The zeta potential of CALS was evaluated in the range of several pHs and given in Figure 5. With the increase in the pH, the zeta potential was decreased. The point of zero charges (pH_PZC_) for the CALS was measured and it was found to be 4.1. The surface of the CALS attains a positive charge if the solution pH is lower than pH_PZC_ and chromium exists as HCrO_4_^–^, Cr_2_O_7_^2–^ at the low pH (Gupta and Babu, 2009). Under this condition, chromium anion can be adsorbed by strong electrostatic attraction. When the pH is greater than pH_PZC_, Cr(VI) adsorbed CALS acquires a negative charge and the adsorption of chromium anion decreases as a consequence of the repulsion amongst negatively charged species. Therefore, the optimum pH for the chromium adsorption onto CALS should be less than pH 4.1 which was further confirmed by the effect of pH for sorption of Cr(VI) study.

### 3.2 Effect of solution pH

**Figure 6:**
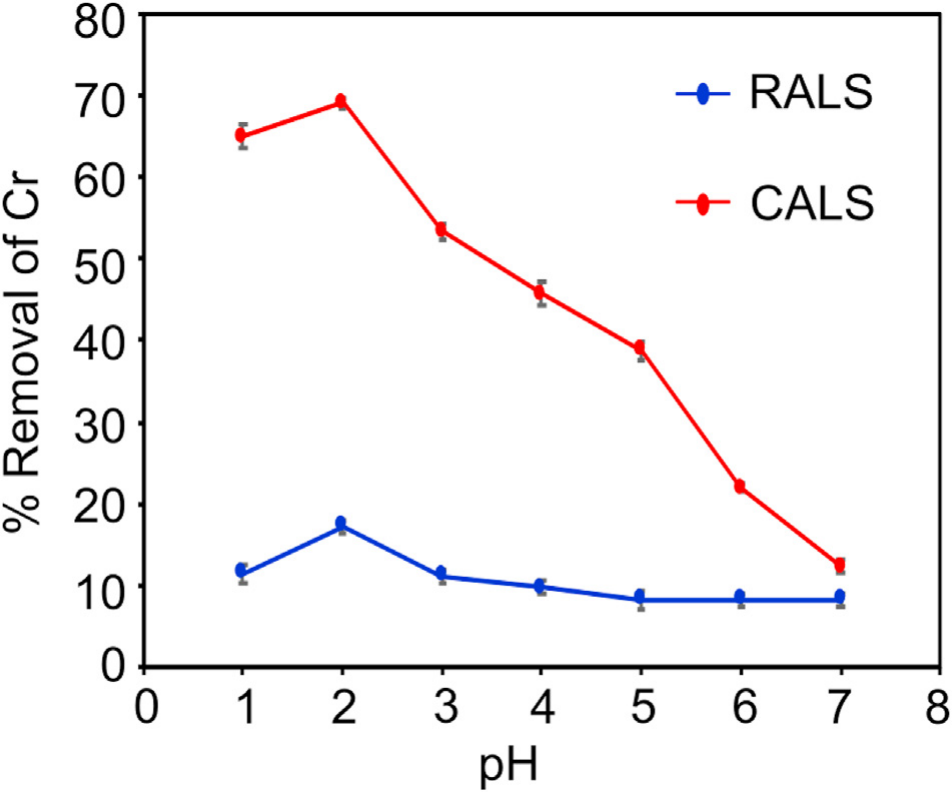
Effect of initial pH for the adsorption of Cr(VI) on RALS and CALS (adsorbent dosages = 1 g/L, Cr (VI) initial concentration = 20 mg/L, contact time = 240 min, temperature = 25 °C).

The effect of solution pH is a crucial factor for the adsorption process. It alters the surface charge of biosorbent, degree of ionization, and specification of the chromium. Protonation or deprotonation of the active site on the sorbent can depend on the pH. Chromium exists as HCrO_4_^–^, Cr_2_O_7_^2–^ on the low pH and exists as CrO_4_^2–^ above pH 7 which is a primary stable species (Gupta and Babu, 2009; Labied et al., 2018). Thus, it was found that the electrostatic interaction between adsorbate and adsorbent during the adsorption reaction is directly affected by variation in pH. Figure 6 shows that pH 2 is optimum for the highest adsorption of Cr(VI), and adsorption of Cr(VI) decreases with further increases in the pH. The percentage removal was increased significantly from 17.33% for RALS to 69.01% for CALS which shows the huge difference between the two adsorbents. Therefore, further study was analyzed only for the CALS. Similar results for the removal of hexavalent chromium by agricultural waste biomass have been reported in prior studies, with pH = 2 being the ideal pH for maximal adsorption (Garg et al., 2007).

When pH < pH_PZC_ (4.1), the CALS surface attains a positive charge, which helps to adsorb the oxyanions of Cr(VI) owing to the strong electrostatic attraction between positive surface of biosorbent and oxyanions of Cr(VI) (HCrO_4_^–^, Cr_2_O_7_^2–^). However, as pH rises beyond pH_PZC_, OH^–^ ions concentration rises and CALS surface charge becomes more negative which leads to electrostatic repulsion between the oxyanions of Cr(VI) and adsorbent negative surface charge. The low Cr(VI) adsorption at pH values higher than 5 is due to competition between OH− and oxyanion of Cr(VI); this leads to repulsion forces between Cr(VI) anions and surface of biosorbents.

### 3.3 Effect of contact time on adsorption of Cr(VI) on CALS

**Figure 7:**
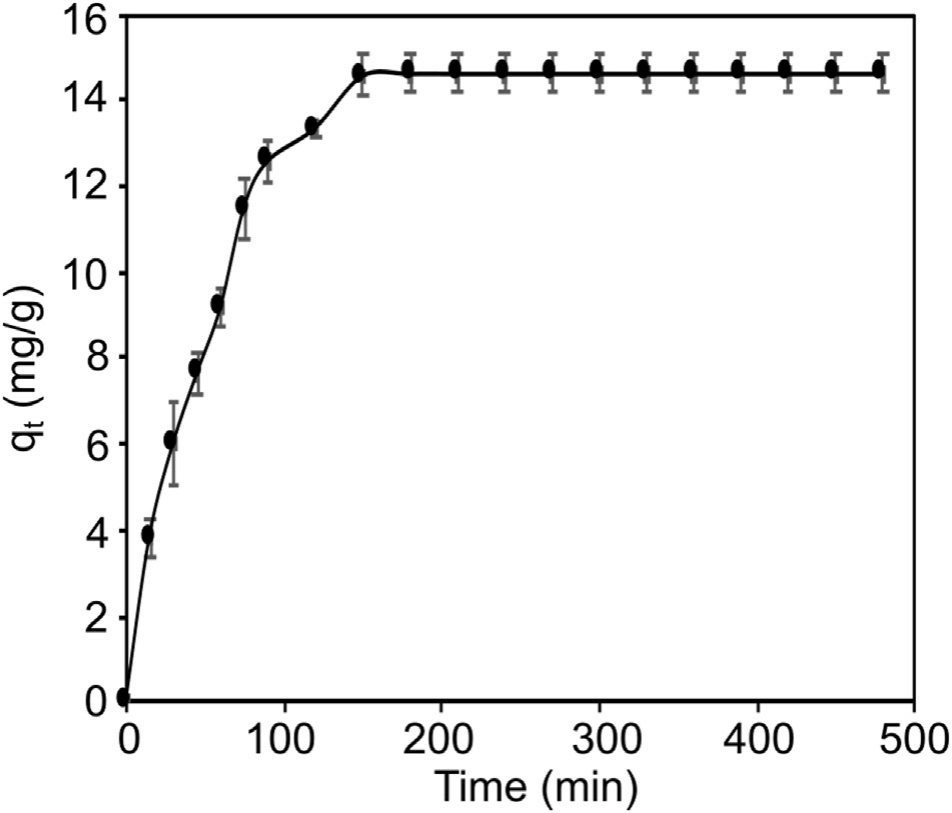
Effect of contact time for the adsorption of Cr(VI) onto CALS (adsorbent dosages = 1 g/L, Cr(VI) initial concentration = 20 mg/L, pH = 2, temperature = 25 °C).

The effect of contact time on the adsorption of chromium on CALS was studied by varying the time while keeping other parameters constant (Figure 7). Adsorption of Cr(VI) ion onto CALS increases with an increase in time. The initial increment in adsorption was found which is due to the availability of many vacant sites at the initial state. As the adsorption proceeded further, adsorption sites were occupied by Cr(VI) and the equilibrium was attained after 180 min. According to the literature, the high adsorption rate at the start of the process is due to many accessible adsorption sites and a high adsorbate concentration, but the adsorption rate gradually reduced as these sites were gradually occupied (Mahmoud 2020).

### 3.4 Adsorption kinetics studies

**Figure 8:**
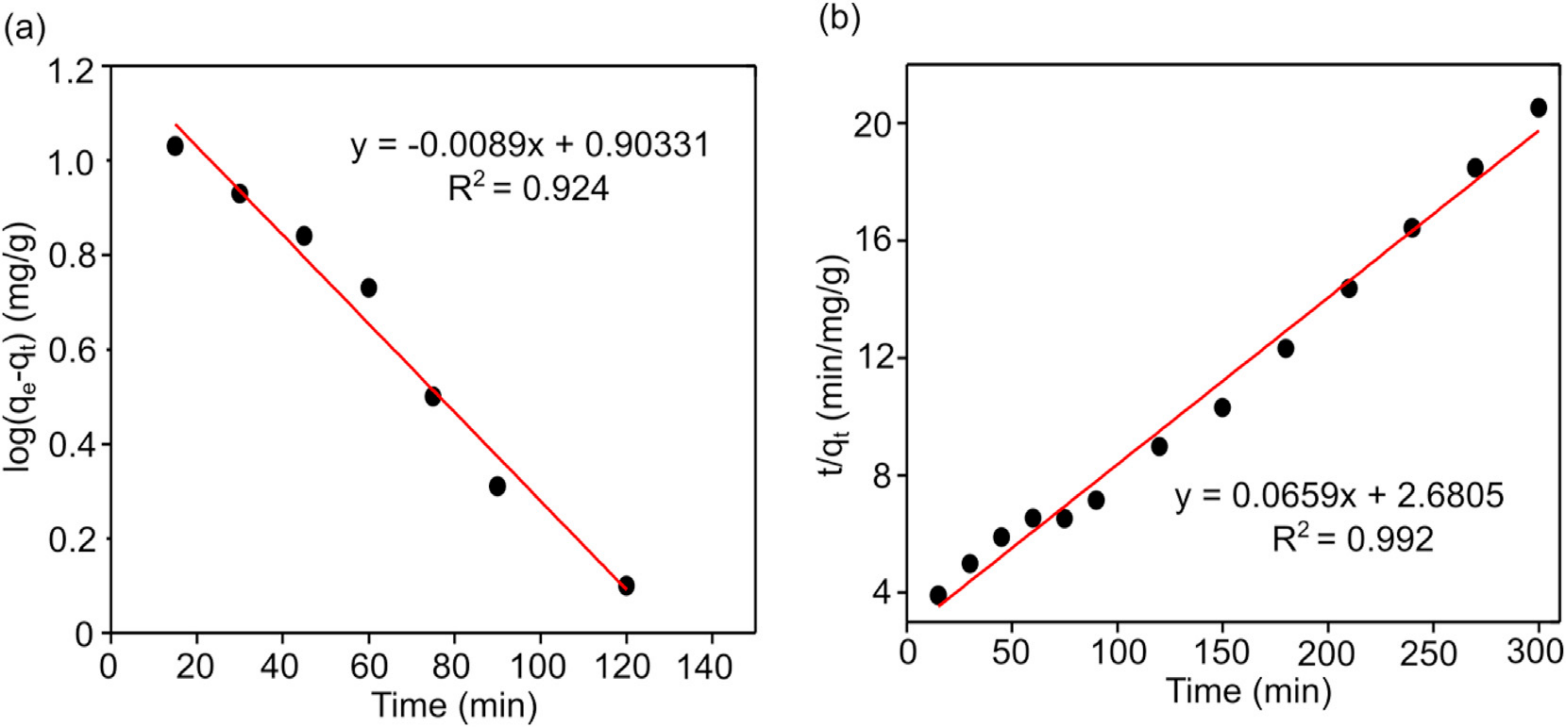
(a) Pseudo-first-order kinetic plot and (b) pseudo-second-order kinetic plot for adsorption of Cr (VI) on CALS.

To determine the kinetics for the adsorption of Cr(VI) onto CALS, pseudo-first-order and pseudo-second-order kinetics models at optimum pH 2 were used to examine the best fitting kinetic model (Figure 8). It was found that the correlation coefficient (R^2^) value for pseudo-second-order was higher than pseudo-first-order for CALS and the calculated values of kinetics parameters are shown in Table 1. Furthermore, in the pseudo-2nd-order kinetic, the theoretical equilibrium adsorption capacity (qe (cal) = 14.61 mg/g) of CALS is close to the experimental value (qe (exp) = 15.71 mg/g). As a result, the Cr(VI) adsorption processes on CALS followed pseudo-2nd-order kinetics, implying that Cr(VI) adsorption is chemisorption-driven. Several researchers have observed comparable results when utilizing biosorbents for removal of hexavalent chromium (Prabhu et al., 2020; Pillai et al., 2013).

**Table 1:** Kinetic parameters for the adsorption of Cr(VI) on CALS.

Order	Adsorbate	R^2^	q_e_(cal) (mg/g)	q_e_(exp) (mg/g)	K_1_ (min^−1^)	K_2_ (mg/g/min)
Pseudo first	Cr(VI)	0.924	14.61	8.59	2.0 × 10^−2^	-
Pseudo second	Cr(VI)	0.992	14.61	15.17	-	1.51 × 10^−3^

**Figure 9:**
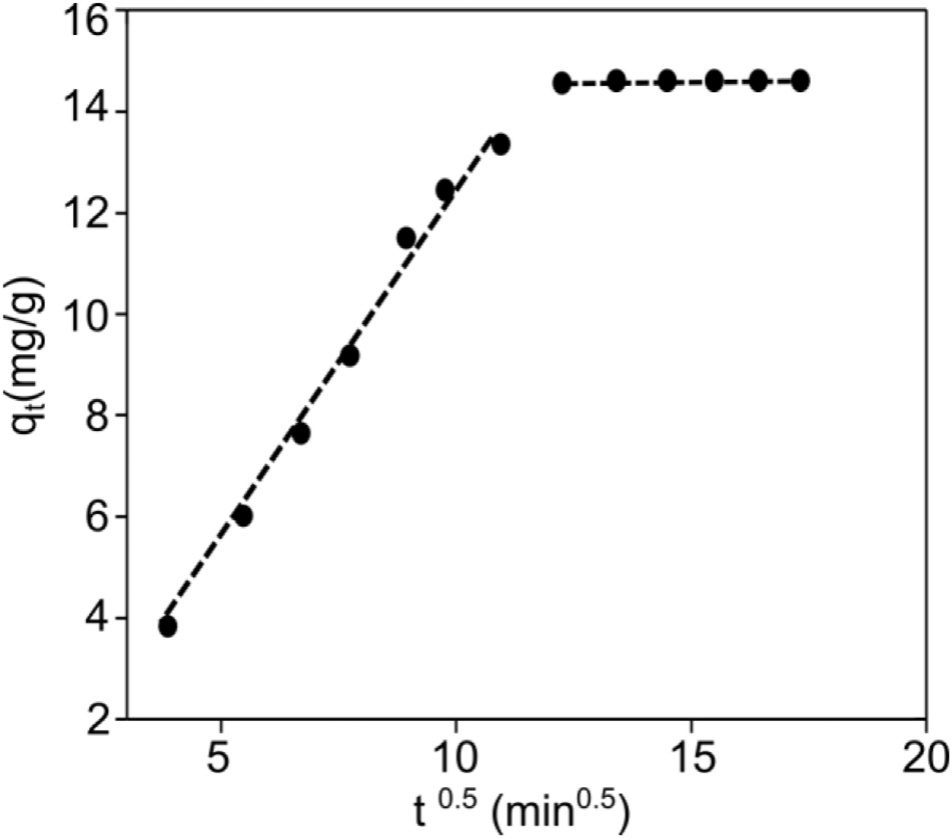
Intraparticle diffusion model for biosorption of Cr (VI) on CALS.

The kinetic models discussed above cannot determine the diffusion mechanism and the rate-limiting step in the adsorption process. Intra-particle diffusion dominates the adsorption only when the plot of q_t_ vs. t^0.5^ is linear. If the plot intersects the origin, then the rate-determining process is because of intra-particle diffusion. The plot with multi linearity (Figure 9) represented that the two or more steps influenced the biosorption process, and intraparticle diffusion wasn't only the rate-controlling step but also by other mechanisms (Mahmoud, 2020; Pholosi et al., 2020). The fitted model shows two linear portions (Figure 9), suggesting two stages in the biosorption process. The first stage is film or external diffusion, in which Cr(VI) ions are transported from the wastewater to the biosorbents' external surface, and the second stage is intra-particle or pore diffusion, in which Cr(VI) ions are diffused from the surface into the biosorbents' pores. However, because the plot does not cross the origin, it is reasonable to assume that the intraparticle diffusion was not the only rate-controlling step in the biosorption process. The equilibrium period for Cr(VI) biosorption onto CALS was around 180 min (t^0.5^ = 13.41 min^0.5^).

### 3.5 Adsorption isotherm model

**Figure 10:**
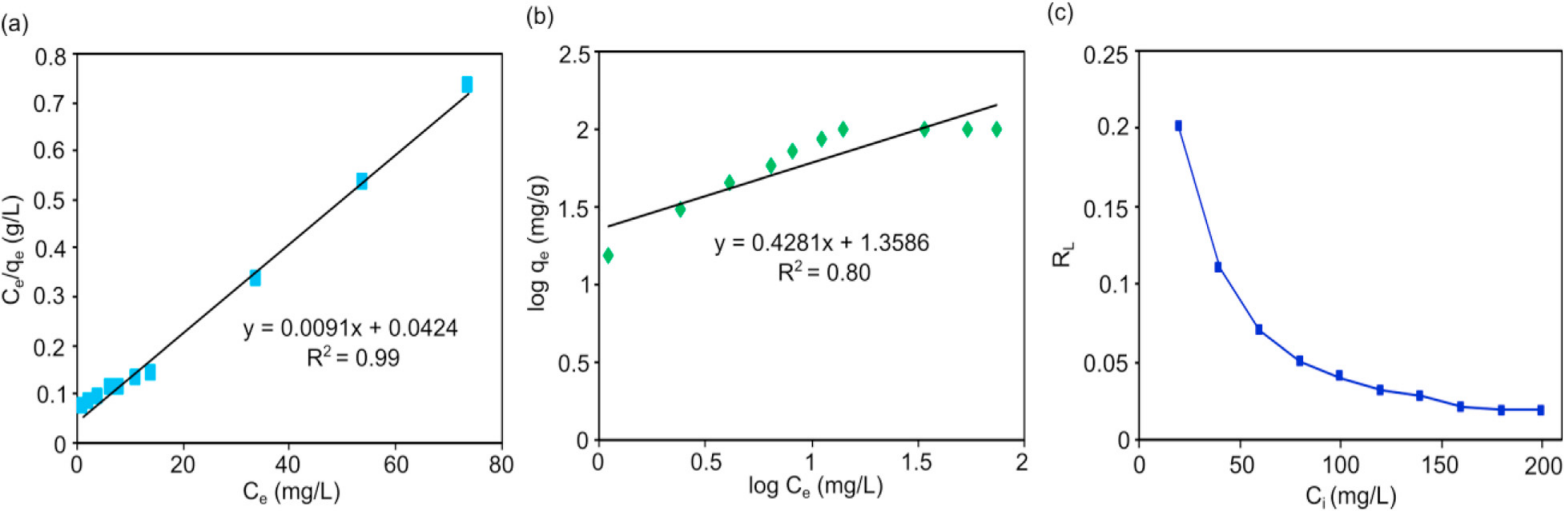
(a) Langmuir isotherm plot (b) Freundlich isotherm plot for adsorption of Cr(VI) onto CALS and (c) variation of R_L_ with initial Cr(VI) concentration (adsorbent dosages = 1 g/L, Cr(VI) concentration = 20–200 mg/L, pH = 2, contact time = 360 min, temperature = 25 °C).

The ability of CALS to adsorb Cr(VI) ion was further determined from the adsorption isotherms plot. The equilibrium data were applied to the Langmuir isotherm model and, Freundlich isotherm model. The outcomes of the plots were used to determine the best fitting model for adsorption of Cr(VI) on CALS. The linearized Langmuir and Freundlich isotherms for CALS are presented in Figure 10(a), and Figure 10(b), and the determined isotherm parameters are tabulated (Table 2). From the isotherm study, it is found that the R^2^ value of CALS for the Langmuir isotherm plot is found to be 0.994 which is greater than R^2^ value for Freundlich Isotherm. The maximum adsorption capacity (q_max_) was found to be 109.89 mg/g using the Langmuir isotherm model. So, we can conclude that the adsorption process dominantly fits the linear Langmuir isotherm. This showed the homogeneous distribution of active sites on the adsorbent surface and monolayer adsorption. Further, the feature of the Langmuir isotherm was explained by the analysis of R_L_. It is found that the R_L_ values were from 0.03 to 0.2 (Figure 10(c)) for all the tested concentrations. So all the values are between 0 and 1 (0 < R_L_ <1) which suggests that the CALS is favorable for the Cr(VI) adsorption.

**Table 2:** Isotherm parameters for the adsorption of Cr(VI) onto CALS.

Adsorbate	Langmuir Isotherm			Freundlich Isotherm		
	q_max_ (mg/g)	K_L_ (L/mg)	R^2^	K_F_ [(mg/g) (L/g)^1/n^]	1/n	R^2^
Cr(VI)	109.90	0.21 × 10^−2^	0.99	22.83	0.428	0.80

The q_max_ of Cr(VI) using CALS investigated in this study is compared with the various other biomass-based adsorbents reported in the various literature (Table 3). The result shows that the Cr(VI) uptake capacity of CALS is satisfactory among the reported adsorbent, suggesting that the CALS can be a potential candidate for the treatment of water samples contaminated with Cr(VI) ion.

**Table 3:** Comparison of Cr(VI) sorption capacities of various sorbents with CALS.

Name of adsorbent	pH		Sorption capacity (mg/g)	Reference	
Tamarind hull	1		81	Verma et al. (2006)	
H_2_SO_4_ treated pomegranate peel	3		28.28	Abdel-Galil et al. (2021)	
Peanut shell	2		4.32	Ilyas et al. (2013)	
H_3_PO_4_ activated sugar beet bagasse	4		52.8	Ghorbani et al. (2020)	
H_3_PO_4_ activated apple peels	2		36.01	Enniya et al. (2018)	
Green moringa leaves	2		3.3	Timbo et al. (2017)	
Litchi chinensis peel	2		50	Ali Khan Rao et al. (2012)	
Diethylenetriamine treated walnut shell	3		50.1	Li et al. (2020)	
NaOH treated rice husk	2		34.85	Mullick et al. (2018)	
Gliricidiasepium Leaf Powder		2	35.71		Suganya et al. (2019)
CALS		2	109.89		This study

### 3.6 Effect of adsorbent dose for the Cr(VI) adsorption

**Figure 11:**
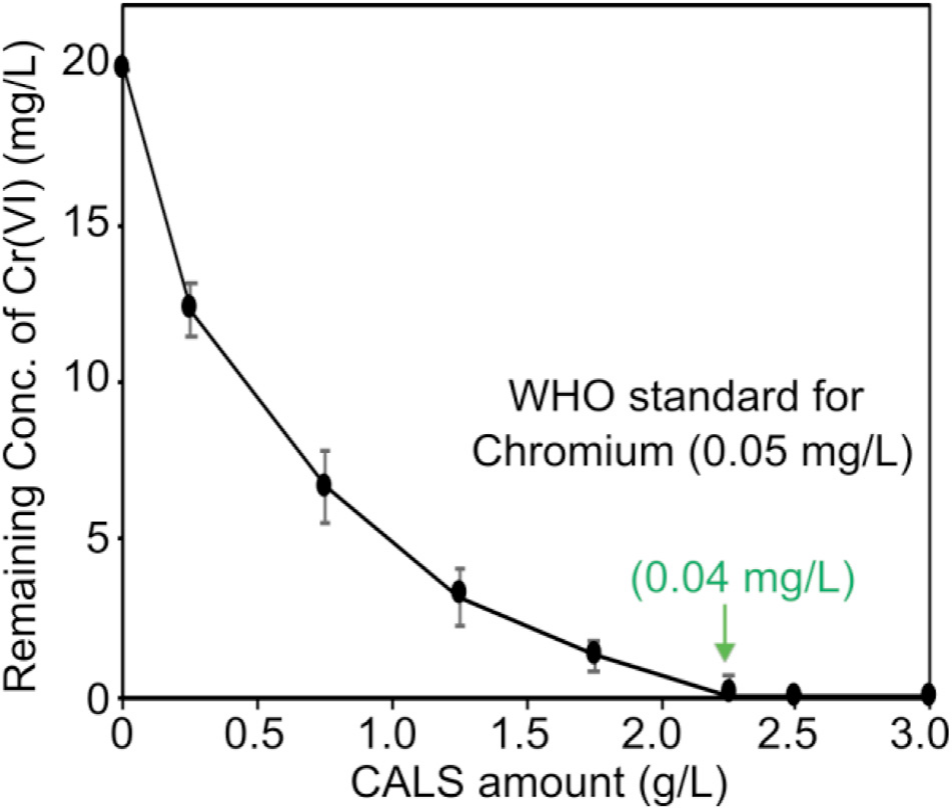
Effect of adsorbent dose on the adsorption of Cr(VI) onto CALS (initial Cr(VI) concentration = 20 mg/L, contact time = 360 min, temperature = 25 °C).

The effect of the adsorbent dose on biosorption activity plays a crucial part in the effective removal of Cr(VI) from aqueous solution and it was examined by changing the amount of CALS as shown in Figure 11. This shows the relationship between remaining Cr(VI) concentrations at the various solid-liquid ratio. According to the WHO standard, the chromium concentration in the drinking water is 0.05 mg/L. Our result suggested that the remaining concentration of chromium can be decreased from 20 mg/L to 0.05 mg/L by using only 2.25 g/L of CALS. Further increase in the adsorbent amount leads to complete eradication of chromium from the water solution. With the increase of adsorbent dose, there is an increase of active sites which is results in the enhancement of Cr(VI) removal efficiency. Hence CALS can be an efficient biosorbent for the treatment of chromium ions from the water solution.

### 3.7 Desorption study and reusability of CASL

**Figure 12:**
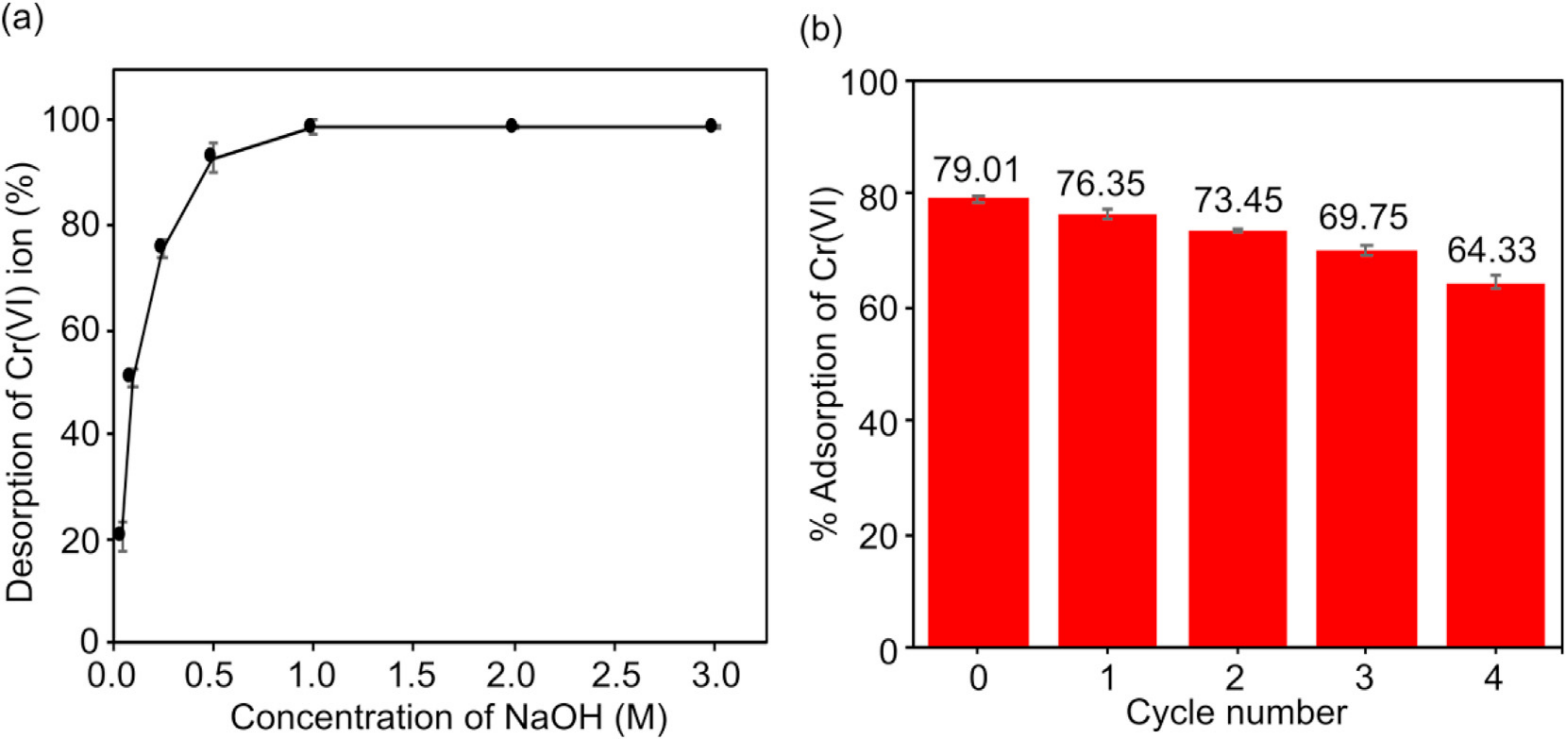
(a) Desorption % of Cr(VI) versus molar concentration of NaOH and (b) variation of the adsorption capacity of the CASL in the subsequent adsorption-desorption cycle.

Cr(VI) is readily adsorbed on CALS at lower pH but poorly adsorbed at higher pH, as demonstrated in findings in effect of solution pH (Figure 6), indicating that elution of adsorbed Cr(VI) may be easily performed by using the weakly alkaline solution. An alkali solution can be used for the regeneration of consumed adsorbent and adsorbed chromate ions. In the alkali solution, hydroxyl ions show a ligand exchange reaction with the adsorbed chromate anions (Pourfadakari et al., 2017). The desorption capability of Cr(VI) adsorbed CALS by using different concentrations of NaOH ranging from 0.05–3.0 M is presented in Figure 12(a). The desorption of chromium increases from 0.05 M (20.32%) to 1.0 M (98.78%) NaOH concentration and it remains constant with further increase in NaOH concentration showing 1.0 M NaOH is the optimum concentration for the desorption process. The increase in desorption might be due to the replacement of adsorbed Cr(VI) ions by the hydroxyl ions released from the NaOH solution until the optimum 1.0 M NaOH concentration. At this concentration 98.78% chromium ions are desorbed and with further increase of the NaOH concentration, there are not enough remaining chromium ions to be desorbed. Additionally, higher NaOH concentration might also played a role to destroy the chemical integrity of the CALS so further desorption remains constant.

The regeneration studies of the prepared CALS were assessed by subjecting four series of adsorption/desorption cycles (Figure 12(b)). Cr(VI) sorption capacity of CALS was decreased by only 15.67% after four cycles suggest that the CALS can be reused at least three times before decreasing the chromium ions uptake efficiency. Desorption of chromium ions under alkaline conditions causes the adsorbent surface to become negatively charged Cr(VI) oxyanion, which allows desorption of metal ions from the biosorbent. Hence, the adsorbent showed the potential to be reused for another cycle without disturbing the chemical stability of the adsorbent. Hence, the effective adsorption capacity with good metal ions recovery and reusability is shown by the CALS.

### 3.8 Mechanism of adsorption and desorption of Cr(VI) onto CALS

**Scheme 2:**
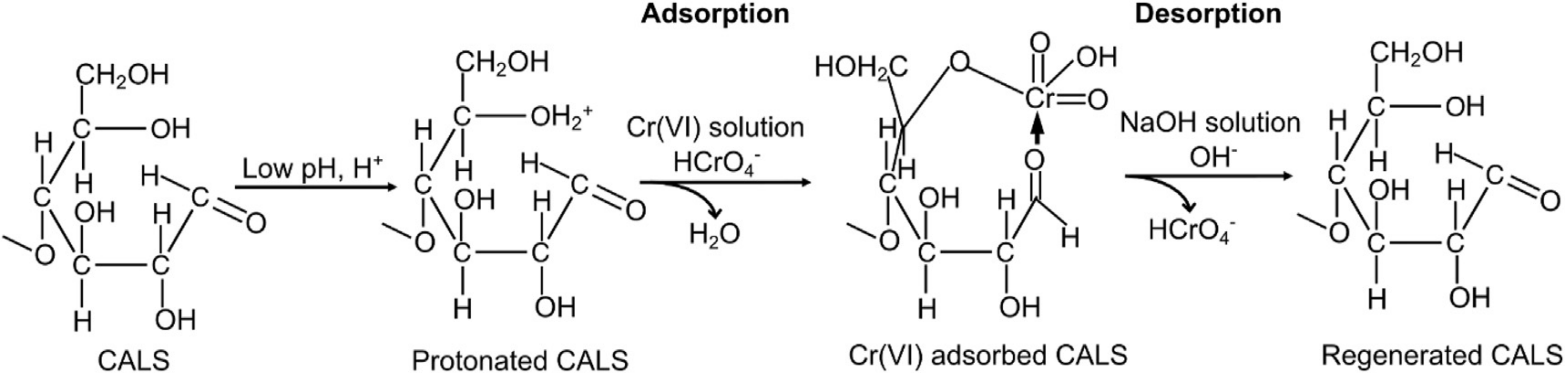
Adsorption and desorption mechanism for Cr(VI) onto CALS.

The adsorbate structure and the surface functional groups of the biosorbents are both important in the biosorption mechanism. The major species in equilibrium in the pH < 6 are HCrO_4_^−^ and Cr_2_O_7_^2−^ (Mahmoud, 2020). When pH < pH_PZC_ (4.1), the CALS surface attains a positive charge, which helps to adsorb the oxyanions of Cr(VI) owing to the strong electrostatic attraction between positively charged functional groups of biosorbent and negatively charged Cr(VI) ions. The mechanism for Cr(VI) adsorption and desorption onto CALS is shown in Scheme 2. At optimum pH 2, HCrO_4_^–^ is the primary form of hexavalent chromium hence, a huge amount of H^+^ ions can be found in the solution at this pH. Therefore, adsorbent forms a positively charged surface due to the process of surface protonation. As a result, the electrostatic attraction phenomenon occurs between protonated surface of adsorbents and HCrO_4_^–^ which leads to its adsorption onto the surface of CALS.

## 4 Conclusions

The present study has shown the widely found Areca leaf sheath can be easily activated into carbonaceous material by its treatment with concentrated sulphuric acid for the efficient remediation of Cr(VI) from water. Adsorbents were successfully characterized by various tools naming FTIR, XRD, SEM, and EDS. The efficacy adsorbent was carried out by using batch process by several parameters such as solution pH, contact time, and adsorbent dosages. The q_max_ of CALS was found to be 109.89 mg/g at optimal pH 2.0, and an adsorption and desorption mechanism was proposed. It is concluded that the Langmuir model is found to be more applicable, and the process of adsorption fits the pseudo-second-order kinetic model. This research demonstrates that economical and easily available arecanut leaf sheath can be modified by a simple treatment method. Raw biosorbents have a very low affinity for removing Cr (VI). Chemically modified biosorbents, on the other hand, have a comparable or better Cr(VI) absorption capacity than conventional adsorbents. However, because sulphuric acid has an environmental impact, the acid modification approach has drawbacks. This necessitates the development of innovative and cost-effective modification methods of biomass Cr(VI) with less harmful environmental consequences. This research was done in the lab scale using batch methods on synthetic waters. Further research needs to be extended in continuous column mode on the real wastewater to promote large scale application of the biosorbents.

## Declarations

### Author contribution statement

Bishnu Datt Pant: Performed the experiments; Wrote the paper.

Deepa Neupane: Performed the experiments.

Dasu Ram Paudel, Surendra Kumar Gautam, Megh Raj Pokhrel: Analyzed and interpreted data.

Prakash Chandra Lohani: Contributed reagents, materials, analysis tools or data.

Bhoj Raj Poudel: Conceived and designed the experiments; Analyzed and interpreted data.

### Funding statement

This research did not receive any specific grant from funding agencies in the public, commercial, or not-for-profit sectors.

### Data availability statement

Data will be made available on request.

### Declaration of interests statement

The authors declare no conflict of interest.

### Additional information

No additional information is available for this paper.
